# Calcification of thoracic aorta – solar eclipse sign

**DOI:** 10.1186/1757-1626-1-133

**Published:** 2008-08-29

**Authors:** Abhijeet Dhoble, Chethan Puttarajappa

**Affiliations:** 1 Department of Internal Medicine, Michigan State University, East Lansing, Michigan, USA

## Abstract

**Background:**

Calcification of thoracic aorta is very common in old people, especially ones with hypertension. This can sometime be visible on plain chest radiograph.

**Case Presentation:**

We present a case of a male patient who had extensive deposition of calcium in the thoracic aorta.

**Conclusion:**

The relationship between aortic calcification and coronary atherosclerosis remains contentious. Computed tomography of the thorax can display this calcification which appears like 'solar eclipse'.

## Background

Calcification of thoracic aorta is very common in old people, especially ones with hypertension. This can sometime be visible on plain chest radiograph. Thoracic computed tomography (CT) can be used for risk stratification for coronary artery disease, and amount of calcium correlates strongly with the prognosis [1].

## Case presentation

**Figure 1:**
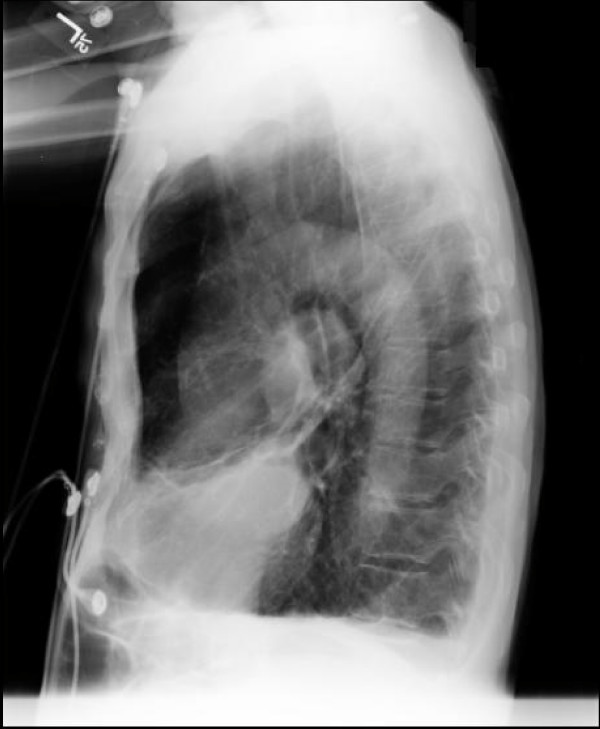
Chest radiograph displaying extensive calcification of the aorta.

**Figure 2:**
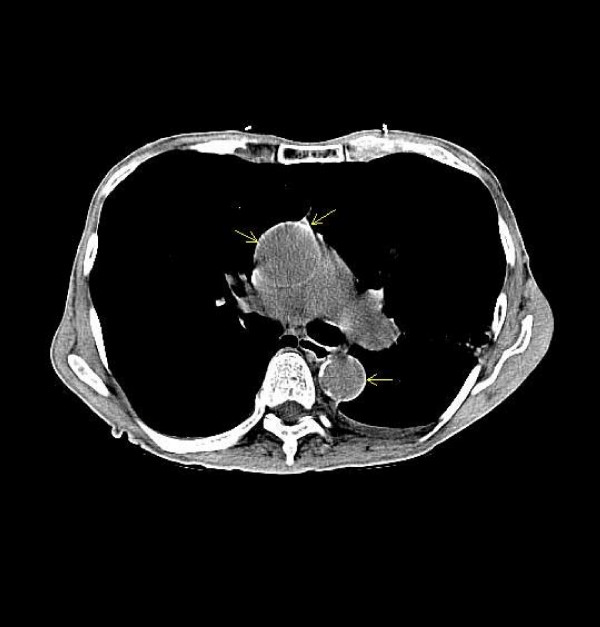
**Computed tomography of the chest showing extensive calcification of ascending and descending aorta.** This classically looks like a 'solar eclipse'.

A 79 year old male with 60 pack year smoking history, emphysema, and hypertension presented with dyspnea and cough. He denied any history of diagnosed coronary artery disease or peripheral artery disease. Chest radiography revealed emphysematous changes (figure 1), along with a coincidental finding of severely calcified thoracic aorta. Computed tomography confirmed these findings (figure 2). It further revealed infected bullae. He was treated with antibiotics, and was discharged with a plan for outpatient cardiac workup.

Patient was not inclined to further pursue in-patient workup on this issue due to other co-morbid conditions. Unfortunately, he was lost to follow up after his discharge from the hospital.

## Discussion

The relationship between aortic calcification and coronary atherosclerosis remains contentious. One study has shown a strong relationship between extra-coronary plaque in the aorta and coronary artery disease [2]. *Bogalusa Heart Study *showed that the severity of asymptomatic coronary and thoracic aortic plaques increase with age and number of cardiovascular risk factors. This study showed strong relationship between aortic plaque formation and smoking [3]. Our patient had extensive history of smoking in the past. Calcium deposition is one of the many processes involved in the atherosclerotic plaque formation, and hence the radio-opaque appearance. This is a complex disease process, and may begin early in childhood [4,5]. Two landmark autopsy studies on soldier's have also proven the fact that the young adults can have advanced coronary artery disease and evidence of aortic plaques [6,7].

Patients with severe aortic calcification may need further investigations based on their cardiac risk factors. If a physician is encountered with chest radiography and CT scan findings shown in these images which were requested for a different issue, further work up must be sought out at the discharge based on individual risk factor profile, and treatment should be individualized.

Figure 2 in our report display classic calcified aortic wall. This appears like 'solar eclipse'. In solar eclipse, the rim of sun is usually visible. The center is visually blocked by the orbiting moon. The appearance in the accompanying CT is exactly similar. The clear area depends on the thickness of the aortic wall. We propose that this appearance can potentially be called as 'solar eclipse sign', which essentially indicates circumferential calcification of the aortic wall.

## Competing interests

The authors declare that they have no competing interests.

## Authors' contributions

Both authors contributed equally in collecting patient data, chart review, and editing medical images. Both authors read and approved the final manuscript.

## Consent

An informed consent was obtained from the patient for publication of this case report and accompanying images in *cases journal*. A copy of the written consent is available for review by the Editor-in-Chief of this journal.
